# Exploring Resilience Among Survivors of Domestic and Intimate Partner Violence in Rural Canada

**DOI:** 10.1111/cars.70033

**Published:** 2026-03-28

**Authors:** Cathy Holtmann, Mayme Lefurgey

**Affiliations:** ^1^ Department of Sociology University of New Brunswick Fredericton Canada

**Keywords:** domestic homicide, domestic/intimate partner violence, informal support, resilience, rural, safety, social ecological model, survivors

## Abstract

In this article, we explore survivor strategies for safety and the development of resilience in the context of domestic and intimate partner violence (D/IPV) experienced in rural Canada. Through the thematic analysis of 24 qualitative interviews, this paper identifies several ways that survivors sought safety on individual, relational, and community levels. Engaging a social ecological understanding of resilience, we highlight how the close‐knit nature of rural cultures presents opportunities and challenges when seeking safety for survivors of D/IPV. The findings of this study inform broader academic and policy discourses on rural D/IPV as well as D/IPV service provision in rural communities by deepening an understanding of the unique strategies for safety of rural survivors and the role of informal support in their resilience as they navigate extreme forms of D/IPV in rural Canada.

## Résumé

Dans cet article, nous examinons les stratégies mises en œuvre par les survivantes pour assurer leur sécurité et développer leur résilience dans le contexte de la violence domestique et conjugale (D/IPV) subie dans les zones rurales du Canada. À travers l'analyse thématique de 24 entretiens qualitatifs, cet article identifie plusieurs moyens par lesquels les survivantes ont cherché à se mettre en sécurité aux niveaux individuel, relationnel et communautaire. En nous appuyant sur une conception socio‐écologique de la résilience, nous mettons en évidence comment la nature très soudée des cultures rurales présente à la fois des opportunités et des défis pour la recherche de sécurité des survivantes de D/IPV. Les résultats de cette étude alimentent les discours académiques et politiques plus larges sur la D/IPV en milieu rural ainsi que sur la prestation de services liés à la D/IPV dans les communautés rurales, en approfondissant la compréhension des stratégies de sécurité propres aux survivantes rurales et du rôle du soutien informel dans leur résilience alors qu'elles font face à des formes extrêmes de D/IPV dans les régions rurales du Canada.

## 1 Introduction

In this article, we describe qualitative findings from our analysis of interviews from the Canadian Domestic Homicide Prevention Initiative with Vulnerable Populations (CDHPIVP) with survivors of high‐risk and severe domestic and intimate partner violence (D/IPV) in rural^1^ Canada. A focus on rural survivors is necessary given the higher severity and risk of violence in rural areas and the increased barriers when accessing formal and informal supports for D/IPV (Anderson et al. 2014). Our study identifies how survivors sought safety, exercised agency and cultivated resilience while navigating incidents of emotional, psychological, physical, sexual and financial abuse perpetuated by an intimate partner or within a domestic, family context. In focusing on survivors’ strategies for safety, we explore a survivor‐centred approach to broader interventions (Goodman et al. 2009). This approach takes their agency seriously, understanding it as a central contributor to resilience in contexts of heightened vulnerability to being killed. Definitions of resilience vary widely, but we conceptualize resilience sociologically as it develops through a process of interaction between individual and collective components (Werner‐Wilson et al. 2000). An individual woman can deal with an abusive and/or potentially lethal relationship when she develops the capacity to harness psychological, social, and material resources for safety available in her local context with the support of others. Drawing from Michael Ungar's ‘social ecology of resilience’ (Ungar et al. 2013, p. 351), we focus on the development of resilience among survivors of D/IPV as a dynamic process of adapting, resisting and transforming resources and relationships in rural contexts. We do not consider survivors in the sample as more or less resilient. We instead explore the various ways in which survivors’ processes of developing resilience were supported by informal and formal D/IPV supports, identifying survivors' strategies for seeking and maintaining safety on individual, relational and community levels.

## 2 Theoretical Framework and Background

### 2.1 A Social Ecological Model of Resilience

In this study, we engage a social ecological model of resilience to better understand and contextualize survivors’ strategies for seeking safety from potentially lethal D/IPV in rural Canada. A social ecological model of resilience (Ungar 2013) assists us in developing a deeper understanding of the unique interplay of individual agency and social support when navigating situations of D/IPV. The model considers psychological, social, and material resources at the individual, relational, and community levels. In a social ecological understanding of resilience, all social levels are equally important in the development of resilience, and there are multiple reciprocal relationships between levels (Ungar et al. 2013, p. 357). In terms of our operationalization of the model, we analyzed factors that related to a survivor's personal identity and history that influenced their responses to D/IPV at the individual level. Sometimes these are referred to as protective factors (Ungar 2011), which might include traits such as extroversion, self‐confidence, sense of self‐worth, emotional regulation, connection to spirituality, commitment to breaking the cycles of violence, goal orientation, academic success, internal locus of control and easy temperament (Alaggia and Donohue 2018). Relationship‐level factors that influence a survivor's experiences of D/IPV, such as support networks among friends, family and neighbours, are influenced by community‐level factors, which, in turn, were analyzed as societal‐level factors (WHO 2002). We considered community‐level factors that contribute to the development of survivor resilience to include access to community resources and services, access to safe places outside the home, and cultural resources.

Moreover, we note that researchers working within feminist theoretical frameworks have long argued that experiences of D/IPV are rooted in issues of power and control (Johnson and Dawson 2011; McPhail et al. 2007). As such, we conceptualize individual power, or agency, as always relational (Koggel et al. 2022). Agency ‘is a property of a system of social relations, a shared resource that can be activated from many different positions within that system’ (Johnson 2005, p. 31). Survivors of D/IPV in rural contexts use their agency to harness informal relationships alongside available formal social resources in unique ways to resist perpetrators of violence and abuse.

When it comes to the lives of survivors of D/IPV, although intersecting structural inequalities have consequences in terms of inequitable access to public services (Fong 2010), this does not mean that survivors do not have agency. The fact that we refer to them as survivors suggests that they have harnessed available individual and collective resources and opportunities to protect themselves from potentially lethal violence (Dunn 2005). Survivors do not survive solely because they are individually resilient. Surviving involves drawing on support at multiple levels of society. Ungar (2011) emphasizes four principles necessary in analyzing a social ecology of resilience. First, we need to decentralize the individual survivor and focus on the social and physical ecology of a particular context. ‘[I]n higher risk environments, resilience is more dependent on the availability and accessibility of culturally relevant resources than individual factors’ (Ungar 2011, p. 6). Second, protective processes between social resources and individuals facing adversity are complex and vary over time. Social resources and individuals change, and some changes can increase rather than mitigate adversity for survivors of D/IPV. Third, certain social environments, like rural areas, may facilitate atypical coping strategies. Survivors’ ‘accurate reading of the environment is a necessary trait when environments fail to facilitate’ safety and are dangerous (Ungar 2011, p. 8). Fourth, we must keep in mind that the development of resilience is culturally embedded. D/IPV interventions designed to support survivor safety and autonomy may take on different meanings in rural cultures.

Informal social support networks and access to both informal and formal resources for the safety of survivors of D/IPV within their local contexts are key to building resilience (Goodman et al. 2022). High rates of domestic homicide in Canada (Dawson et al. 2018) are an indication that our collective response to D/IPV is insufficient because we are not adequately supporting individual and relational efforts in seeking safety. We argue that women who survive potentially lethal D/IPV in rural areas of Canada are resilient because of their capacity to harness their individual resources and navigate the few social supports available to them. Better understanding the factors that contribute to the resilience of rural survivors of D/IPV can contribute to recommendations for improving social support systems.

### 2.2 Contextual Factors for Vulnerability to D/IPV in Rural Canada

In Canada, rates of domestic violence perpetrated by spouses or former spouses and family members are higher in rural areas than in urban areas (Northcott 2011). Residence in a rural location is also a risk factor for domestic homicide victimization, and the risk of both lethal and non‐lethal violence increases significantly when D/IPV survivors have recently left a relationship or are in the process of leaving (Burczycka 2022). Between 2011 and 2021, police reported 1,125 gender‐related homicides of women and girls in Canada. Two‐thirds of these homicides (66%) were perpetrated by a current or former male intimate partner, 28% by a male family member, 5% by a man known to the victim and 1% by a stranger (Sutton 2023, p. 6). In 2021, the rate of gender‐related homicide was 2.5 times higher in rural compared to urban areas of Canada (Sutton 2023, p. 3). The number of women victims of domestic homicide in Canada rose dramatically from 53 in 2023 to 81 in 2024 (Statistics Canada 2025).

Several economic and social factors contribute to the prevalence of D/IPV in rural communities, including higher poverty and unemployment rates, uncertain economic conditions and industry instability (e.g., communities reliant on resource extraction, impacts of climate change), deindustrialization and/or seasonal work trends (Jeffrey et al. 2019; McDonald 2011; Saxton et al. 2021). Patriarchal and conservative rural culture and the relationship between these ideologies and the use of firearms also play a role (Annan 2008; DeKeseredy 2021; McDonald 2011; Musielak et al. 2019; Jeffrey et al. 2019; Saxton et al. 2021). Other factors, such as lack of cell phone coverage, challenging climates and terrain, geographic and social isolation and lack of public transportation are also characteristic of rural D/IPV cases and have been identified as obstacles to accessing support in the literature over the last two decades (e.g., Biesenthal et al. 2000; Jeffrey et al. 2019; Youngson 2020).

The intersecting lived experiences of a survivor must be contextualized as rurality often exacerbates other life conditions, such as un/under employment and the lack of access to medical and other services (Kohtala et al. 2023). Wood et al. (2024) found that rural survivors of D/IPV often face situations of coercive control which involve threats to their pets, larger companion animals and livestock. Indigenous women are at a higher risk of experiencing severe D/IPV than non‐Indigenous women, and this reflects the long history of colonial violence in Canada, the consequences of intergenerational trauma, and systemic racism. ‘Barriers to accessing services may disproportionately impact Indigenous women residing in remote communities’ (Heidinger 2021). Kaye and Glecia (2025) argue that the ‘criminal legal system’ response to violence against Indigenous women sustains ongoing colonial gendered violence in Canada. Racialized immigrant women survivors of D/IPV also experience significant challenges in accessing public services including lack of professional interpretation, differing understandings of D/IPV, the complexities of the intersection between D/IPV and immigration policies and processes, a lack of trust between service providers and immigrant women (Holtmann and Rickards 2018) and isolation from cultural communities in urban centres (Panchuk et al. 2024). In their analysis of the experiences of women survivors of D/IPV living in rural Saskatchewan, Soares et al. (2024) also highlighted difficulties experienced by those identifying as LGBTQ. For example, they found a lack of trust in service providers based on the intersecting stigmas associated with being LGBTQ and a survivor of D/IPV, as well as the denial of shelter services to transgender women (Soares et al. 2024).

## 3 Data and Methods

The CDHPIVP from which our sample of survivors is drawn, ran from 2015 to 2022 and focused on identifying emerging risk assessment, management and safety planning strategies for victims of D/IPV from four vulnerable populations: Indigenous, rural, remote and northern, immigrant and refugee and children exposed to D/IPV. As part of the CDHPIVP, interviews were conducted with key informants such as frontline service providers and representatives from D/IPV not‐for‐profit organizations and shelters, in addition to the one‐on‐one survivor interviews discussed in this paper. Preliminary insights from the analysis of this data highlighted the complex and intersecting barriers affecting populations facing violence in rural, remote, and northern areas of Canada (Saxton et al. 2021). Key informants who work directly with survivors in rural areas reported that geographic location and isolation were problematic in addition to factors such as lack of public transportation, higher rates of poverty, distance from services or other sources of help, and a general lack of available services (Kohtala et al. 2023; Saxton et al. 2021; Youngson 2020; Youngson et al. 2021). Patriarchal and cultural norms and beliefs characteristic of rural communities were also noted as concerning, including the normalization of gun culture and outdated beliefs about women's roles within the home and society (Saxton et al. 2021). Concerns around confidentiality when accessing services or reporting violence were also highlighted (Kohtala et al. 2023; Saxton et al. 2021; Youngson 2020).

To address the intricacies of rural D/IPV, key informants made recommendations such as the need for highly contextual safety planning that considers factors such as a gun on the property and distance from neighbours or police (Saxton et al. 2021). They spoke about the complex intersections of challenges at systemic, organizational and client levels. They emphasized the need for increased communication and collaboration among rural agencies and their urban counterparts, including improvements to referrals, outreach services and risk‐assessment frameworks (Youngson et al. 2021). Key informants stressed the need for increased awareness of rural concerns within the sector and more broadly within society, as well as more culturally affirming programming options to address the gaps in identifying, intervening, and ending cycles of D/IPV in rural regions (Saxton et al. 2021; Wuerch 2020; Youngson 2020). Our analysis of interviews conducted with rural survivors of D/IPV echoes these reported concerns and trends.

Ethics certificates for conducting interviews with survivors of D/IPV and close friends or family of domestic homicide victims, according to the standards outlined in the Tri‐Council Policy on Ethical Conduct for Research Involving Humans (TCPS2), were sought and obtained by researchers associated with the CDHPIVP from 13 Canadian universities (including the authors’ university) and research licenses were sought and obtained from Yukon and Northwest Territories. Eligibility criteria for survivor interviews included having experienced potentially lethal domestic violence between 2006 and 2016, being 18 years of age or older and currently safe from D/IPV. Participants were recruited through press conferences, social media campaigns, media scans, direct emails to service providers and by word of mouth through various research and community‐based networks. After expressing interest in the research, potential participants received a detailed letter of information about the project. Research coordinators obtained verbal consent to conduct short screening interviews to determine eligibility, to ensure the participants’ safety and access to support, establish rapport and inform them of the nature of the study and the interview process. Interviews were conducted after obtaining verbal consent from participants, mostly virtually, by trained graduate research assistants between 2019 and 2022 using a trauma‐informed, narrative approach to interviewing where survivors were asked to share their story, with a focus on actions and strategies to keep themselves and/or their families safe. Additional methodological details are available on the CDHPIVP website and in Straatman et al. (2022).

As part of the CDHPIVP, 129 interviews were conducted with survivors of D/IPV and individuals who were close to victims of domestic homicide. A group of co‐investigators, postdoctoral fellows and graduate research assistants worked to classify and conduct preliminary coding of the interview transcripts to ensure that data from each of the vulnerable populations would be analyzed. Based on our expertise concerning access to services for D/IPV survivors from two vulnerable groups (rural and immigrant), we purposively selected the sample of English‐speaking, non‐Indigenous survivors of D/IPV from rural areas for our analysis. Our intention was to focus on one group alongside other researchers engaging with this data set and exploring the unique barriers impacting rural survivors. We believe our theoretical framing is appropriate for analyzing settler transcripts, but other framings are more appropriate for the Indigenous survivors (e.g., Kaye and Glecia 2025) from rural areas. Our choice to analyze data from a relatively homogenous subsample ensured that our findings provided a richly detailed and nuanced description of an under‐researched population. Importantly, the contexts specific to Indigenous survivors within rural settings are largely imposed by displacement through colonization and are both different and distinct from largely farming‐based settler culture(s) in rural areas. We feel that our analysis functions in conversation with other researchers associated with the CDHPIVP.

This article describes the findings of our analysis of a sample of 24 semi‐structured qualitative interviews with English‐speaking, settler women‐identifying survivors who experienced, or were a child witness to high‐risk and severe D/IPV between 2006 and 2016. These participants lived in rural regions at the time the violence occurred and were asked to share their stories about how they sought safety and/or reduced risk when faced with a range of harmful behaviours, including all forms of physical, sexual, emotional, spiritual and financial dimensions of violence, abuse and control (CDHPIVP 2019).

Participants were from various regions across Canada^2^ and diverse age and socio‐economic groups. Two participants identified as immigrants, and 20 had children. The average age was 40 years old. Most of the abuse experienced by interviewees was committed within an intimate relationship (e.g., dating, common‐law, spouse, long‐term partner), yet, in some cases, the interviewee spoke from the perspective of reflecting back on their childhood of witnessing D/IPV against a parent and/or their own experiences of violence from a parental figure or family member.

Interview recordings were transcribed verbatim and de‐identified. The authors analyzed the transcripts from survivors thematically (Braun and Clarke 2006), coding deductively to identify broad characteristics of experiences of D/IPV to contextualize our analysis and to identify if the data analyzed speak to or differentiate from trends reported in the broader research on rural D/IPV in Canada. The social ecological model of resilience (Ungar 2011) guided our analysis and helped us to identify the strategies and resources used by rural survivors of D/IPV when seeking safety by classifying survivor strategies in three broad categories: individual, relational, and community levels of safety‐seeking—choosing to rely on the social location of resources as an organizing principle. Within these three categories, we grouped together like‐strategies to form sub‐themes (e.g., prevention, diffusion, fleeing, safety planning). In terms of process, the authors each coded a subset of transcripts and met periodically to discuss the themes found to ensure reliability, rigor and consistency in our coding process. This allowed us to identify any discrepancies or possible sub‐themes that were overlooked. After engaging in this iterative process, we then each reviewed all transcripts once more to ensure consistency and evidence for the themes we describe in this article. Lastly, we highlighted recommendations identified by survivors to ensure that our recommendations are survivor‐driven and rooted in lived experience.

In terms of exclusion, we did not analyze societal‐level factors. Societal‐level factors impact all survivors of D/IPV, albeit differentially due to intersecting social structures. Although Canadian policies and funding for services remain inadequate to address survivor needs (Tastsoglou et al. 2022), feminist researchers and activists have succeeded in convincing governments that D/IPV is a serious and widespread social problem warranting a National Action Plan to End Gender‐Based Violence (FPT 2022). While this is a positive step, it remains necessary to better understand how rural survivors of D/IPV make use of their personal and relational resources and the social supports available to them at the community level as they seek safety from violence.

## 4 Findings

Mapping onto what has been shared above in the broader literature, our analysis found that D/IPV takes place in ways that are both unique and complex in rural Canadian communities. One of the key elements discussed by survivors that made their situation particularly vulnerable was the degree of isolation, geographically but also socially, that they experienced navigating D/IPV in a rural context. Their isolation was oftentimes a central part of the abuse and control they experienced—further complicated by life conditions such as a lack of financial independence (e.g., generational farm ownership) or a lack of technological access (e.g., poor internet access or cell service). Many lived on large properties or farms with firearms or other weapons on‐site, which amplified fears of death or severe violence. Some survivors had few neighbours and lived in disconnection from a sense of community or from their family and other relationships. Participants in our sample described severe D/IPV experiences including sexual assault, broken bones, strangulation, suffocation, a knife held to the throat, held captive in a vehicle driven recklessly at dangerously high speeds, bullets shot at the house, attempts to burn down the house, sexual trafficking, branding, threats of suicide by the abusive partner and pets shot or killed, and coercive control. Many of the perpetrators of severe violence did so while intoxicated by drugs and alcohol.

Survivors reported a lack of local D/IPV services and difficulty accessing a D/IPV shelter and medical care, or experiencing long wait times for emergency services to arrive during a violent incident. More so, the lack of specialized D/IPV‐specific training within local services such as policing, hospital, and schools was frequently discussed. Beyond the availability or quality of services, many survivors were afraid to report the abuse they were experiencing out of concern for a lack of confidentiality or neutrality of service providers, given their interconnected and small communities. Against this backdrop of intersecting challenges, the survivors interviewed had various individual, relational and/or community‐level strategies for seeking safety from a violent partner or parent.

### 4.1 Individual Level Strategies

Survivors described an array of adaptive strategies and intentional actions strategically taken both to diffuse violence in acute scenarios and to prevent the onset of violence. Survivors exercised agency by adapting to various risks and challenges in a wide range of scenarios to protect themselves, their children or their friends and family more broadly.

Survivors used negotiation or distraction strategies to diffuse or deflect situations of violence, such as redirecting the conversation or using negotiation skills to convince the abuser not to choose violence or to de‐escalate during an incident. One survivor reported using conflict mediation and diffusion skills to help calm her abuser down: ‘I would use my skills as a social worker to kind of help calm him down… He is a very disturbed person because of his own past and his own struggles. His mental base… I would do everything possible to not be that trigger’ (Survivor 23, Central). Another survivor used distraction to have her abuser occupied while she got the items needed for her escape:

So I literally – I had – I'm like, “Oh could you bathe the kids, could you do that just for me? Just so I can get things ready?” and he agreed to it. And I literally took the diaper bag and I put as many diapers in and as many clothes of theirs that I could… I literally had the clothes on my back and maybe an extra shirt. Like, nothing more. I grabbed their passports, I grabbed their – I had some jewelry that belonged to my mom who has passed, that I just wanted to grab those things… “I put them in the truck, and that's all I had.” (Survivor 25, Atlantic)

Survivors changed their behaviour and daily routines to avoid contact with their abuser, both during the abusive relationship and following separation. In some cases, this happened within the home, such as locking themselves in a room to avoid violence or seeking safety in numbers, such as spending time with siblings, pets or family members inside the home. For example, a survivor whose husband had guns in the house and on the property shared, ‘I barricaded myself in my bedroom at night… I pushed furniture up against the door and I slept with a crowbar underneath my mattress… At one stage I had a dog, a big dog and the dog used to sleep in the room with me. That was comforting and I had two sons and while they were at home I felt a lot safer (Survivor 2, West Coast).

In other cases, strategies to avoid an abuser involved spending a lot of time outside the home at work, school or other community activities, and going on walks with their dog or children. Many increased their time spent outside the home with informal support networks, including friends or family members. One survivor explained how she would go to her mother‐in‐law's house, as the abuser would not want to go, and if he did go, he would not behave abusively in front of his family: ‘Well, it felt like a break, right? Like I could take the kids over and she would help me with the kids and he wouldn't. Like he either wouldn't come, so we could just get away from him, or he wouldn't behave that way at his mom's house’ (Survivor 13, West Coast). Survivors' individual‐level strategies for seeking safety also included the development of safety and escape plans. This included always knowing where key items are in the home or vehicle, taking discreet or inconsistent routes when driving, changing vehicles or having pre‐identified hiding or safe areas to run to, including spaces such as a room with a lock, a vehicle with the key inside, a wooded or discreet area or a neighbour's home to flee to. Survivors reported having their keys, phones, and other personal items ready to get out quickly if needed. Survivors also sometimes hid a go‐bag or cash in their home or car in case they needed to flee. Survivors used technology, such as turning on the GPS tracker on their phone, so that family could find them if they went missing. One survivor who had an eight‐year relationship but never lived together with her abuser described her strategies:

I found new routes to come home because he would sometimes just be here and not tell me. So, I would you know, drive in a separate way or would have to drive right by my house but I would see if his vehicle was there. Um, you know, I hid keys in trees, so that I could either get in or out quickly if I needed to. (Survivor 24, Central)

Another interviewee's strategy involved having a wooded area to run to in the night to hide from her abuser to call 911.

Following the end of a relationship, survivors had various strategies to avoid their abuser and feel safe, such as moving to a busy street, to a city with more resources or into secure buildings with neighbours close by. A female survivor and police officer shared, ‘I was finally able to move. Like two months later I got my own place and I got on the busiest street in town though I felt safe because I knew at any time I could run into the street and ask for help versus when I lived next door to him and was in the middle of nowhere’ (Survivor 10, Central). Survivors frequently reported moving to new towns or living arrangements that would be discreet from their abuser pending a separation, taking refuge in a shelter or living with a security system in their new home. In many cases, survivors gave up their homes and belongings in the pursuit of safety. A former vice president of marketing for an international company shared, ‘That was when I packed up a tractor trailer overnight. I put all my stuff in a tractor trailer, put the tractor trailer in storage, put my kids in the car and we took off and we left our home. We just walked out and left our home and that was hard. We started all over again’ (Survivor 4, Central).

### 4.2 Relational Level Strategies

Rural survivors of potentially lethal D/IPV spoke about how they interacted with family and friends—people in their existing relational networks who became part of their informal support system—in ways that supported their safety. Here we describe how they confided in family and friends to enhance safety. At times, people in informal support systems connected survivors with the formal D/IPV services. Some survivors made safety plans with people in their support networks, and several used technological tools for staying safe.

Survivors had mixed feelings about telling their families about what they were going through. While they appreciated and needed emotional and pragmatic support, they did not want their families to worry about them. A survivor from a fishing community explains:

[My family members] were a good support, yes, they were very good to me and my kids. If it wasn't for them, I don't know where I'd be. But I didn't want to – I didn't want them to have to be worrying about all this stuff. I worried that, you know, they'd have to worry. I worried about them worrying about me… And I didn't, I didn't – how can I put this – I didn't like to get them involved until I absolutely had to, but most of the time that the domestic violence happened, I had to leave quickly, and they had to be involved, or there was police involved. (Survivor 1, Atlantic)

Some survivors worried that reaching out to their family for support would put their loved ones in danger. Others made the distinction between disclosing to members of their family of origin but not to the family of their abuser. They explained that some families ‘took sides’ when they had information about D/IPV. Survivors had different experiences of family relationships and had to decide whether they could trust family members to support their resilience and whether they wanted to put this burden with its potential risk on their families.

More frequently, the survivors talked about their friends’ support. An example comes from a survivor who relied on a core group of friends while in a relationship with a violent partner. During a phone call, a woman from this group used their friendship to put a bit of pressure on the survivor to seek help from the formal D/IPV service sector:

She's like, “Well if I give you a list of resources, can you get in touch with one of them for me and tell them what you told me?” I was like, “Yeah sure,” like mostly doing it because I love my friend and I'm just like, “Look, you know, you've been kind enough to listen, I'll do it just so that you feel better,” because I didn't want her to worry, right? So then that's when I called the shelter a couple days later… if she didn't say that, I never would have gotten in touch with them. (Survivor 19, Prairies)

The survivor appreciated her friend's kindness in listening to her experiences of severe violence and reciprocated by calling a shelter. This contrasts with friends who respond with fear and outrage and unwittingly add to survivors’ anxiety. Some survivors will seek safety for the sake of their relationship with friends, rather than for themselves. This illustrates the power of supportive informal relationships in building resilience amongst survivors of D/IPV.

Sometimes support from friends was pragmatic—they provided safe places of refuge from abuse. A survivor of physical and verbal child abuse by her father did not tell anyone about what was happening at home for fear that it would make things worse. She describes her strategy for safety:

I spent as much time out of the house as possible. I spent a lot of time with other people my age, my friends from the neighbourhood that I grew up with and their families. I never did reach out for help. But that definitely did make me feel safer at least at the time. And even just knowing that if something happens, I can go to [a friend's] house or [another friend's] house or whatever. It was walking distance. (Survivor 9, Prairies)

In reflecting on these experiences, she believes that the parents of her friends knew that her father was ‘a notorious drunk’ but no one asked about her home life, they simply offered her a safe place. She believes that hers was a positive strategy, and she felt supported by her friends’ families.

In another example, a survivor spoke about being in a relationship for years before she learned from staff at a sexual assault centre that she was experiencing sexual violence. Eventually, her partner hurt her physically (strangulation, broken bones). She turned to a friend for help when victim services would not respond to her request for support:

Once I had started with the sexual assault center the very first thing that I had done when I was making arrangements, I had phoned victim services who couldn't support me or wouldn't support me because I hadn't been involved in the justice system; I hadn't pressed charges; police hadn't been involved. They weren't any help to me at that point. [I] reached out to a friend and so from there, that was the first – my first contact with anybody about what I was going through with my friend. And she was actually the one that testified in court which supported him going to jail so, so that was kind of the first step of, of being able to speak about what I was going through. (Survivor 24, Central)

In some provinces, survivors of D/IPV can access support from government‐funded victim services only after the abuser has been charged by police and they have been deemed a victim of crime. This support can include financial compensation and access to mental health services. Yet most survivors of D/IPV do not contact the police, and if they do, they want the police to stop a violent incident rather than charge their partner with a crime (Barrett et al. 2011; Ursel 2006). Unable to access the formal support she needed to stay safe in the process of ending the relationship, the survivor talked to her friend about the violence she was experiencing before contacting the RCMP. It was the combination of support from staff at the sexual assault centre (formal support) and confiding in her friend (informal support) that contributed to this survivor's resilience and the eventual incarceration of her partner.

Survivors shared information about making safety plans with their family and friends. Several of these involved the use of technology. After leaving a violent marriage, a mother of two children talked about the code word she would text her brother if her husband showed up.

No one knew about [the violence], I didn't start talking about it until a week before I left. I finally told my brother… So once I started talking about it, it became real and I got really anxious and really scared, and within a week I had moved out. I had taken the kids and came up with a plan, and I came up with a safety plan along the way. You know, if I ever texted my brother my mom's name, that was our code word, that he was to call the police right away and to send them out. (Survivor 25, Atlantic)

Another survivor set up her mobile phone to send an SOS signal during a violent incident, along with a pin drop on a map to her friend, so they could call the police with her location.

A research participant who married her partner shortly after graduating from university, shared that the relationship had been emotionally abusive from the start. Then one night he strangled her. Friends urged her to leave him, but she felt dependent on him—she had moved to a small town to build her life around his. She had reached out to the local women's shelter and had read research on domestic violence. She credits an online network of strangers for convincing her of the gravity of the situation:

I spent a lot of time looking online, like Reddit which is a website where everyone can post about anything. And I was reading about abuse – and it's like an online forum, right, so it's not articles, it's people talking. And uh, somebody posted in a thread saying “People who have been with partners who have hurt them – has there ever been a chance where he's only hurt you once? Like he's only put his hands on you once and you've been together for years”. And that was literally it. So I'm reading through it, and there's like 100 responses and all of them are like – because here I'm still thinking “Well, he'd never do this again. It's been six months, if it was going to happen again it would have.” Also not thinking that I'd been walking on eggshells and stuff. So all the responses are like “No, no, no, no, no!” and I'm like “Oh.” and there's only two that said he hadn't done it again… It was [reading] other people's stories [that helped me to act]. Because I knew, like in my head, all the – you know, I've read papers and read articles – I knew all the research on domestic violence. But obviously if that [knowledge] was going to do something, it would have done something a long time ago. So, I needed to read other people's stories who had been there and what they did. (Survivor 19, Prairies)

In this example, the survivor knew intellectually how dangerous her relationship had become, but she ‘didn't want to hurt him’ and hoped that he would stop acting violently. She credits other survivors’ online stories for convincing her to report the violence to the police.

### 4.3 Community Level Strategies

Survivors often chose individualized and relational strategies for safety due to the lack of D/IPV‐specialized services in their rural areas. When services did exist, participants in the study were sometimes reluctant to access them due to the perception that confidentiality and privacy would not be upheld, putting them at increased risk of violence. Survivors expressed concern about knowing a service provider personally or fearing that ‘the whole town would know’ what was going on in their personal life if they sought help. Because of this, survivors frequently reported feelings of shame and isolation when navigating experiences of D/IPV in rural communities. For example, one survivor reported that of the 220 residents in her community, 75 were direct relatives of her abuser, leaving no one safe to turn to (Survivor 11, Central). In another example, a woman went to file court papers related to her abuse and realized that it was her abuser's friend working at the courthouse. The friend relayed that she was there to support the abuser, which left the survivor feeling alone and afraid that public service providers in her town, who should be neutral, had taken sides and breached confidentiality:

And when I filed my papers of course it's my ex's friend that takes—she's working at the courthouse and then she phones him right away and says, “Guess who was just here?”… And then this court employee would also during trial sit with him on his breaks and console him say, “Don't worry you'll be partying soon enough,” loudly so I could hear it right? So, it just, it became everybody was related, it felt like and I felt very alone in the—in that small town. I didn't grow up there and he did. (Survivor 8, Central)

While the relationship survivors have with community‐based services is varied in rural communities, several did seek out community‐based services, either locally (when available), or in a nearby city centre. In many cases, members of informal support networks referred survivors to community agencies, and workers in community agencies linked survivors to other services, providing a more holistic approach to care for some. Most notably, survivors sought safety with the assistance of staff at D/IPV shelters, their employers, D/IPV outreach programs or related specialized services, local healthcare providers, private counsellors, government agencies like victim services, the police or a trusted but not D/IPV‐trained service provider (e.g., a local mom's group, school staff member) in the community.

When D/IPV shelters/second‐stage programs were available and accessed, the experiences of survivors were overwhelmingly positive. Shelters offered non‐judgmental support, validation and opportunities for survivors to connect and be part of peer support groups with others navigating similar experiences. Several survivors reported that shelter workers were their first point of contact in the D/IPV sector and the first people to understand what they were experiencing. This was critical for many, especially for those who were still questioning if their experiences were abuse and for those who did not have supportive family or friends who understood the complexities of D/IPV. Shelters were a key part of survival for many participants and contributed to their resilience when navigating unthinkable realities. Shelter workers helped link survivors to vital services such as counselling or legal aid, or provided them with items they needed or had to leave behind, such as clothing, toiletries or childcare items. One survivor described her experience:

They gave me a car seat—they were just—they were fantastic every step of the way… They got my kids into play therapy… they got me into free counselling services with family service [municipality], they just everything, they got me in touch with legal aid right away knowing that with my maternity leave income I would be approved. They got me in touch with a list of lawyers that provided legal aid to meet with in [city]. (Survivor 4, Central)

Shelters provided survivors with safe housing (e.g., with security cameras and alarms) as well as assistance with forming safety plans, which were vital for survival in some cases:

It worked really well with me just to keep me safe, because he would, he would show up at any time, like, at other apartments that I had. He would just show up – sitting on the end of my bed one night. So there [at the shelter] I felt safe. Even though he did try to get in there, and they did arrest him. But I still felt safe because of the alarms, let you know that somebody's there. (Survivor 1, Atlantic)

Some survivors engaged their employers and employees at their workplaces as part of their safety plan, while at work or more broadly. For a survivor employed in a health care setting, her strategy for staying safe at work involved all staff being aware of what the abuser looked like and choosing a safe word that would notify people in her workplace if she was in immediate danger:

What they did is they put a picture of him at the front desk so if he came in the door they would be notified…Sometimes he wouldn't let me get to work or I wouldn't be able to get out to get to work, if I called in sick, if I was in danger, I'd give them a certain word. All the – they had a meeting where all the nurses knew all this information, so if I said a certain word then they would know to call the police right away. (Survivor 1, Atlantic)

Survivors shared that their employers played a key role in supporting their resilience by accommodating time off for childcare, appointments, legal proceedings and linking them to community services and counselling support.

Some survivors sought support and safety through other community‐based services that were not D/IPV specific but played a vital role in their journey to safety. For example, one survivor confided in a counsellor at a post‐partum support group who helped connect her to other services where she made a formal safety plan and eventually left her abuser (Survivor 11, Central).

Lastly, in terms of community‐based services, we want to briefly highlight the varied interactions reported with law enforcement. Most survivors reported unhelpful interactions with RCMP or police officers, yet a few said that officers had specialized training in D/IPV, and this was impactful. One survivor shared how a police officer understood the complexities of abuse, did not judge her for wanting to return to the abuser and offered strategies for her to cope with her emotions when seeing him in court: ‘She [D/IPV‐trained police detective] was the best. And I remember her standing in front of me and she said, “Do not even make eye contact. Don't even look in his eyes. Look at me if he looks at you, you don't look back you just keep eye contact with me”’ (Survivor 4, Central). The D/IPV trained police detective helped the survivor and her children access a shelter and various community services. However, when she went back to the abuser, this survivor had negative encounters with other police officers. When her partner involved her in an insurance fraud case as part of D/IPV, she felt she was treated as a criminal and not as a victim when navigating police services (Survivor 4, Central). This survivor's story exemplifies the complexities of police involvement and how critical proper training and education are in addressing the complexity of abuse for all those who respond to cases of D/IPV.

## 5 Discussion

Our analysis of information shared by 24 survivors of potentially lethal D/IPV that took place in rural regions of Canada provides evidence of how the development of resilience includes survivors’ strategies for safety using resources available at the individual, relational and community levels. We found individual‐level strategies for safety included negotiating with and/or distracting the abuser to prevent violence, disrupting routines or moving to avoid contact with the abuser, and having a safety plan during a violent incident. These individual strategies overlapped with survivors’ relationships with family, friends and neighbours, especially when accessing safe spaces outside the home. This theme is aggravated by their heightened vulnerability in rural contexts (Ungar 2011), as many survivors in the sample did not have easy access to a women's shelter and/or specialized D/IPV services. We found that in cases where survivors did not have access to or learned they could not trust family or friends for support in staying safe, they turned to community‐level services for help. Sometimes relational‐level strategies for safety employed by survivors helped build bridges to community‐level services, but in other examples, when community‐level responses were unhelpful, survivors of D/IPV relied on support from friends and neighbours. Our findings show that informal support networks at the relational level were an important resource for the development of rural survivors’ resilience and the success of their strategies to stay safe, regardless of whether they accessed formal services. The relational‐level strategy of using technology to connect with friends, family, or other survivors was important in seeking safety. In line with previous research, our findings show that resilience in the face of D/IPV is a multi‐faceted and complex process depending on a variety of individual, social and contextual factors (Crann and Barata 2016; Ungar 2011). The emphasis on surviving D/IPV as a process of resilience is important because our findings show that survivors used different strategies to stay safe over time, including after the relationship had ended. The process of surviving D/IPV is not linear, because some strategies were more effective than others at different stages of the relationship. Survivors learned to build upon the plans for staying safe that worked. Some of these strategies, like not trusting public service providers, may appear ‘atypical’ but were a product of survivors’ experiences of betrayal and accurate reading of how the close‐knit nature of rural cultures could be weaponized against them (Ungar 2011).

Our findings concerning individual‐level strategies for seeking safety highlighted how rural survivors used their understanding of those who hurt them and their social contexts. Some survivors used their knowledge of the abusers to avoid triggering violent responses or their skills in negotiation to alter or manipulate abusers’ perceptions and behaviours. Crann and Barata describe these strategies as cognitive and behavioural ‘shifts towards resistance’ (2016, p. 860) while Yalcinoz‐Ucan refers to them as resisting disempowering situations (2022, p. 513) and Black et al. (2020) categorize them as acts of everyday resistance. We found that survivors frequently used several strategies to avoid being alone with violent partners. They took their children temporarily away from the person who had harmed them and, in some cases, sought safety in the presence of family, friends and neighbours. Goodman and colleagues refer to this as ‘survival‐focused coping’, one of the aims of which is to keep the survivor and their loved ones as safe as possible (2009, p. 318) in a context of limited resources.

In a review of published articles on help‐seeking for D/IPV, Sylaska and Edwards (2014) found that most survivors are likely to seek out informal sources of support, be that friends, family members, classmates, co‐workers or neighbours and that they consider informal supports to be most helpful. Likewise, in a qualitative study of survivor help‐seeking in moments of acute danger, Goodman et al. (2022) found that although most women are reluctant to seek help, those who did tended to choose family, friends, and neighbours. Our findings at the relational level corroborate the important role that informal networks of support play in the resilience of rural survivors of D/IPV as they seek safety. Like our findings, Goodman et al. (2022) found that the responses of family members to survivors’ requests for help were mixed. In their study, family members who had their own histories of violence were either desensitized to the harms of D/IPV or too traumatized to respond in ways that supported survivors’ strategies for safety. Our findings show that sometimes family members or friends sided with the abuser, and survivors had to be careful about whom they disclosed to or sought help from. Survivors’ caution in seeking help from family and friends was also prompted by concerns for the safety of their loved ones who might be threatened by an abuser attempting to regain control after the disclosure of D/IPV.

Research shows that positive responses from family, friends and neighbours to survivors’ disclosures of experiences of D/IPV include a listening ear, advice, and emotional and practical support (Goodman et al. 2022; Sylaska and Edwards 2014). Our exploration of survivors’ strategies for safety highlighted the importance of practical support from family, friends and co‐workers. In their qualitative study of survivors of D/IPV in rural Kansas, Bosch and Bergen (2006) found that although emotional support contributes to survivors’ well‐being, it will not help in ending abuse. ‘Helpers must assist women in making decisions best for themselves and their children’ (Bosch and Bergen 2006, p. 319). Some of our findings highlight supportive responses from informal networks that sometimes involved individuals acting as a bridge between the survivor and available community service providers. Friends and family encouraged survivors to call the shelter or to contact the police. A friend documented incidents of abuse, which were later used in court. Our findings show that supportive relationships were helpful to survivors as they navigated public services. In explaining why survivors prefer informal supports, Aspinall and Crocker (2025) contrast the relationality of informal supports with the transactional nature of public services for survivors of D/IPV. Some of the survivors in our sample were in violent relationships for years, because it takes time to safely leave a violent partner, and the violence does not end when the relationship does. Relationships with friends and family can support survivor resilience long‐term, while public service interventions end.

Our findings show that the community‐level resources that best sustained supportive relationships with rural survivors of severe D/IPV over time were women's shelters. This aligns with research that emphasizes the ways that staff at women's shelters support survivors’ resilience by providing a place where they feel safe as well as validation, information, peer support, and help in navigating and connecting with other community services (Mantler et al. 2022). Most shelters in Canada provide services, support and programming to residents and non‐residents, in‐person and remotely via phone or online (Women's Shelters Canada 2023). In their study of shelters in Ontario, Wathen et al. (2015) argue that despite persistent underfunding and scrutiny from governments, shelter workers do everything they can to maintain client‐centred services. These services are guided by the understanding that ending a violent relationship and healing from trauma is a process that involves various kinds of services over time (Wathen et al. 2015, p. 139). Research on organizations in rural communities which offer informal support to survivors, like churches, shows that these support networks are more effective if they involve collaboration with local women's shelters (Nason‐Clark et al. 2018). There was evidence in our findings of collaboration between public service providers to support D/IPV survivors at the community level.

Advocates and researchers have long argued that employment is an important component in ending D/IPV by ensuring survivors’ economic security (FPT 2022; Holtmann and Rickards 2018; Panchuk et al. 2024) and our findings show that a supportive work environment contributes to their resilience. Several survivors in our study who had jobs in their rural communities received support from their employers following disclosure of D/IPV, although some felt that their co‐workers did not believe how dangerous the situations were and did not adequately ensure their safety. The results of a national, online survey on domestic violence in Canadian workplaces mirror our findings. The results showed that slightly more than half of the respondents who disclosed their experiences of D/IPV to co‐workers indicated that ‘mostly positive’ things happened. Yet the remainder indicated that there was no response, nothing happened, or a mix of positive and negative things happened (Wathen et al. 2014, p. 8). The *Canada Labour Code* requires federally regulated employers to properly respond to employees’ disclosure of violence and to undergo mandatory domestic violence workplace training (CLC 2025). Many provinces require employers to conduct risk assessments for D/IPV as part of their health and safety plans and have included provisions for D/IPV leave in their employment standards legislation (New Brunswick D/IPV in the Workplace Committee 2025).

Finally, there were very few survivors in our sample who found the police response to their calls for protection from violent partners helpful. This finding is reflected in research, which shows mixed evaluations of the police response to D/IPV survivors. In an analysis of data from the 1999 General Social Survey of Victimization in Canada, D/IPV survivors who called the police reported that police came to the scene and collected evidence for a report and/or investigation in only half of the cases, contrary to mandatory charge and arrest policies (Barrett et al. 2011, p. 53). In a study of police understanding of D/IPV in New Brunswick, Gill et al. (2019) found that female officers were more likely than male officers to understand the complexity of D/IPV. Many officers do not take the complexity of D/IPV into account, nor do they consistently conduct risk assessments to determine the most appropriate response to a survivor's call for help. In a survey of police officers across Canada, also led by Gill et al. (2023), initial results showed that most participants (92%) were able to identify a hypothetical scenario as IPV when it featured physical violence, but only 67% considered a scenario featuring non‐physical violence to be IPV. In rating the importance of well documented risk factors for IPV, they rated factors associated with physical abuse as more important than tactics of non‐physical abuse. Gill and colleagues’ recommendations for more specialized education and training on D/IPV for police align with the findings from our study. Likewise, Saxton et al.'s (2022) analysis of qualitative data from Canadian police interviews highlights inconsistencies in addressing situations of D/IPV and calls for greater collaboration between police and other service providers at the community‐level.

Even though our sample is regionally diverse, it is not representative of all rural D/IPV survivors in Canada, and their rural contexts are not homogeneous. Yet, an important aspect of the rural contexts of the survivors’ experience requires further reflection—rural culture. Sandberg (2013) reminds us that rurality (as a discursive construct) developed in response to the conditions of geographic and social isolation. People in rural areas live further away from one another and from public services and employment opportunities than those who live in urban areas. This presents a paradox because rural survivors of D/IPV must rely on family, friends and neighbours to support their strategies for safety due to the inaccessibility of other options. Although the close‐knit nature of rural communities can contribute to survivor resilience, the lack of confidentiality results in members of informal and formal support networks heightening the risk of domestic homicide by sharing information, intentionally or unintentionally, with the abuser or members of his support network. Our findings highlight the efficacy of individual‐ and relational‐level strategies used by survivors when seeking safety from D/IPV and illustrate the significant role that informal support networks play in the development of resilience. Yet the strategies do not differ substantially from the literature, despite the heightened risk of lethality due to the rural context of their experiences.

## 6 Conclusion

Our findings concerning the social ecology of resilience that undergirds the safety of a sample of survivors of severe D/IPV in rural regions of Canada contribute to the small yet growing body of qualitative research on the experiences of survivors of D/IPV in rural contexts. This rich empirical evidence of the relational, social and contextual contributions to building the resilience of D/IPV survivors helps us better appreciate how individual strategies for seeking safety are connected to and dependent on informal support networks of family, friends and neighbours in addition to formal community supports.

In terms of recommendations, on an individual level, our findings showcase the resourcefulness and resilience of survivors to adapt and seek safety in situations of acute danger. There is an opportunity to strengthen resources for survivors related to individual safety planning, including safety plans for when they are at home with their abusers and need to rely on negotiation or de‐escalation to increase their safety. Individual safety plans need to be realistic and contextualized within the rural landscape realities (e.g., a plan to lock oneself in a safe area while waiting for EMS services; a safe place or a neighbour to run to with supplies). When internet and/or cellular connectivity is available, rural survivors can benefit from increased awareness of how to harness technology to increase their safety both in acute situations and following separation from their abuser (e.g., how to send alerts when in danger to an emergency contact). Survivors may also benefit from increased awareness of free online or app‐based supports such as the newly developed iHeal app that helps survivors in the safety‐seeking process (Ford‐Gilboe et al. 2025).

Our findings shine a light on the large extent to which informal supports are sought in rural communities. The sheer degree to which informal support was sought identifies a need to increase the capacities and awareness of those providing support and an untapped potential opportunity to better engage informal supports either as links to formal D/IPV supports or within the planning and care models offered by formal community supports (e.g., offering consultations to informal supports on how to help survivors, engaging informal supports in safety planning exercises with shelter or outreach workers). Our findings show that there is room for improvement in the quality of support from survivors’ informal relationships through public awareness and education campaigns and resources about D/IPV. These outputs should focus on the danger of lethality of D/IPV, respecting survivors’ agency, the importance of confidentiality, and provide tangible strategies on how to offer ongoing emotional and practical support to survivors (e.g., through non‐judgmental active listening, accompaniment or safety planning). Moreover, our findings reveal the efficacy of survivor‐to‐survivor support during or after D/IPV through peer support offerings (e.g., in shelter programming, online forums and groups).

There is an opportunity for formal supports to better harness peer support opportunities, including those that can connect isolated rural survivors to survivors in other regions. In addition to strengthening the capacities of informal supports in rural communities, we recommend that people who are part of formal support networks in rural areas, like shelter workers, employers and community leaders from various sectors (e.g., education, health, criminal justice) have increased professional opportunities to learn more about the complexity of D/IPV, how to strengthen relationships with D/IPV survivors, and contribute to survivors’ resilience by supporting and building on their individual strategies for safety. This might include increased training and education on best practices in responding to disclosures for community groups.

On a community‐level, service providers must understand what existing resources have been identified as overwhelmingly supportive and those that have had diverse responses from D/IPV survivors, ranging from those that are very helpful to those that contributed to further harm. We found that rural survivors feel most understood and supported by D/IPV shelter and outreach workers. We recommend that shelter staff and outreach workers not only improve the support from survivors’ family and friends through education and information sharing but also be part of facilitating community‐coordinated responses and training of other frontline and/or community leaders and organizations (e.g., healthcare workers, schools, churches, libraries). Many shelters do this already to some extent, but increased funding for them to carry out this work is essential, especially in rural Canada.

We also want to briefly draw attention to the critical role that some employers played in helping D/IPV survivors in rural areas access critical services while maintaining their employment. Advocates and researchers have long argued that employment is an important component in ending D/IPV by ensuring survivors’ economic security, and our findings show that a supportive work environment contributes to their resilience. In practice, this could look like more training for Canadian employers to support survivors in their workplace.

Supportive relationships with rural survivors of D/IPV depend on trust. Because many rural communities are tight‐knit, confidentiality is a critical concern for ensuring survivor safety, especially when family, friends and neighbours of a perpetrator of abuse are also providers of public services. Typical resources for supporting the safety of survivors of D/IPV in rural contexts lack meaning in a culture of patriarchal domination and victim blaming. Rural survivors need options for confidentially accessing services outside their local communities, such as domestic violence outreach, safety planning, counselling, peer support groups or legal services via websites, apps, text messaging and by phone. Because rural technological connectivity is inconsistent and computers and cell phones can be monitored without survivors’ knowledge, we recommend that rural communities explore the idea of creating safe hubs with strong connectivity within existing public spaces, such as school libraries and hospitals. Here, D/IPV survivors could access computers free of charge to look up information on and get access to D/IPV support services, as well as learn safe browsing practices.

In sum, our findings highlight how rural D/IPV survivors adapt, resist and seek safety through creative means on individual, relational and community levels and develop resilience. Our findings show that the strength of survivors’ longer‐term supportive relationships with family, friends, co‐workers and neighbours can be vital and facilitate the process of building bridges between informal supports and available formal D/IPV services in rural communities, if survivors choose to engage with public service providers. Learning from lived experience accounts of what rural survivors did to seek or maintain safety can offer insights into how to enhance formal services and supports, and build on what is working well.

## Funding

The CDHPIVP received funding from the Canadian Women's Foundation and the Social Sciences and Humanities Research Council of Canada.

## Conflicts of Interest

The authors declare no conflicts of interest.

## Endnotes

1 CDHPIVP defines rural as a community or geographic location with a population of less than 10,000 (Jeffrey et al. 2019).

2 Transcripts were identified by the time zones in which interviews were conducted. We identify participant data by region: Atlantic (AST), Central (EST), Prairies (CDT, MST) and West Coast (PST). Participants identified with the Central, Prairies and West Coast regions include those from northern Canada.
